# An Evaluation of Parents’ Experiences of Patient Engagement in Research to Develop a Digital Knowledge Translation Tool: Protocol for a Multi-Method Study

**DOI:** 10.2196/19108

**Published:** 2020-08-04

**Authors:** Alison P Thompson, Shannon E MacDonald, Eytan Wine, Shannon D Scott

**Affiliations:** 1 Faculty of Nursing University of Alberta Edmonton, AB Canada; 2 Department of Pediatrics Division of Pediatric Gastroenterology & Nutrition University of Alberta Edmonton, AB Canada

**Keywords:** patient engagement in research, knowledge translation, functional constipation, digital health resources, patient-oriented research, formative evaluation

## Abstract

**Background:**

The last decade has seen increasing calls for patient and public involvement in health-related research due to an ideological shift toward more equitable methods of knowledge development and an effort to increase the usability and relevance of knowledge by improving outcomes in clinical practice. Patient engagement includes simply informing patients to offering complete decision-making autonomy to individuals, groups, communities, caregivers, friends, and families who have personal experience and knowledge of a health issue. Despite the use of patient engagement methods in research, evaluation has lagged, resulting in a knowledge gap that makes it difficult to foster capacity and sustainability for patients and researchers alike since little is known about how effective patient collaborations in research are built, maintained, or improved. This study centers on pediatric functional constipation, a common condition that affects children and families. Since parents play a pivotal role in treatment, they are an optimal group to engage in improving the resources and support available to them.

**Objective:**

This study aims to use patient-engagement methods to establish a research collaboration with parents to cocreate a digital knowledge translation tool for parents caring for a child with functional constipation and formally evaluate the patient engagement processes within this project to build the science of patient engagement in research.

**Methods:**

Members of the parent collaborator group will be recruited from previous participants who expressed interest in the development of a digital knowledge translation tool. The group will collaborate with the research team to create a tool to address patients’ support and information needs when caring for a child with functional constipation. The parent collaborator group will then be evaluated in a multimethod study design. Data will be digitally and anonymously collected from all members of the parent collaborator group, using the validated Public and Patient Engagement Evaluation Tool (PPEET) patient questionnaire. Descriptive statistics will be used to report group characteristics and question responses. Qualitative analysis will be used to understand open-ended question responses. Specifically, directed content analysis will be used to assess themes of the Patient Engagement in Research (PEIR) Framework with a combination of deductive and inductive analyses. Findings will be integrated into the discussion if there are sufficient commonalities and inter-relationships. The final manuscript will include reporting of each element as described by the Good Reporting of a Mixed Methods Study criteria.

**Results:**

Recruitment is planned for June 2020. Data collection for the evaluation of patient engagement processes will occur upon completion of the digital knowledge translation tool. The results of this study are expected to be published by the end of 2020.

**Conclusions:**

This study will provide valuable information about parents’ experiences participating in child-health research and is a fundamental step in building the science of patient engagement in research.

**International Registered Report Identifier (IRRID):**

PRR1-10.2196/19108

## Introduction

Health research programs have historically been considered the exclusive domain of professional scientists. Whereas families’ experiential knowledge and input in the clinical environment has been prioritized for many years, the research context has been slower to consider patients as contributors to knowledge development. Despite the intention to create clinically relevant knowledge, research programs have continued to develop knowledge in isolation from patient input. Consequently, patients and families have been at the center of a paradox between the ideological positions of clinical practice and research [1], while questions about the usability and relevance of research findings to improve clinical care have persisted. Over the past ten years, there have been increasing calls for patient and public involvement in health-related research. The impetus for this shift is twofold; an ideological shift towards more equitable and less hierarchical methods of knowledge development [2,3] and an effort to increase the usability and relevance of knowledge as evidenced by improved outcomes in clinical practice.

Although terminology varies around the world, in Canada, the terms *patient-oriented research* and *patient engagement* are commonly used in health care, aligning with guidance from the Canadian Institutes of Health Research. Patient engagement is defined as “meaningful and active collaboration in governance, priority setting, conducting research and knowledge translation [3].” Furthermore, the word patient is an umbrella term that includes individuals, groups, communities, caregivers, friends, and families who have personal experience and knowledge of a health issue [3]. Although including patients and families as part of the research team is a fairly straightforward ideal, diversity in operationalization has slowed knowledge development related to effectiveness and best-practices of patient engagement [4-6]. Similarly, evaluation of the processes and outcomes of patient engagement in research has lagged, resulting in a meager evidence base for patient-oriented research [2,4,7-10]. The current lack of evidence regarding patient engagement in research makes it difficult to foster capacity and sustainability for patients and researchers alike since little is known about how effective patient collaborations in research are built, maintained, or improved.

Furthermore, parents are a unique subgroup of the patient engagement population that merits further exploration because of their dual roles, inherently representing both themselves as caregivers and their children as patients [11-13]. Specifically, in this study, we are engaging with parents caring for a child with functional constipation. Functional constipation is a type of constipation that occurs without underlying medical or physiological causes. Prevalence rates amongst North American children are reported in the range of 9%-18% [14], and these patients often have higher rates of emergency department visits and specialist care. Specifically, pediatric functional constipation accounts for upwards of 25% of pediatric gastroenterology visits [15,16]. Parents of children with functional constipation are critical stakeholders in the successful management of pediatric functional constipation because the treatment regime is ideally provided and monitored at home. As such, collaborating with parents of a child with functional constipation offers an innovative approach to ensure clinicians can provide proper support, and parents have resources tailored to their needs. We are engaging with patients in pediatric functional constipation research both to improve clinical care for families and to evaluate parents’ experiences participating in child-health research, as a fundamental step in building the science of patient engagement in research. That is, the patient engagement process is widely applicable, meaning others can use this protocol to guide patient engagement processes and evaluation in any number of study populations.

**Figure 1:**
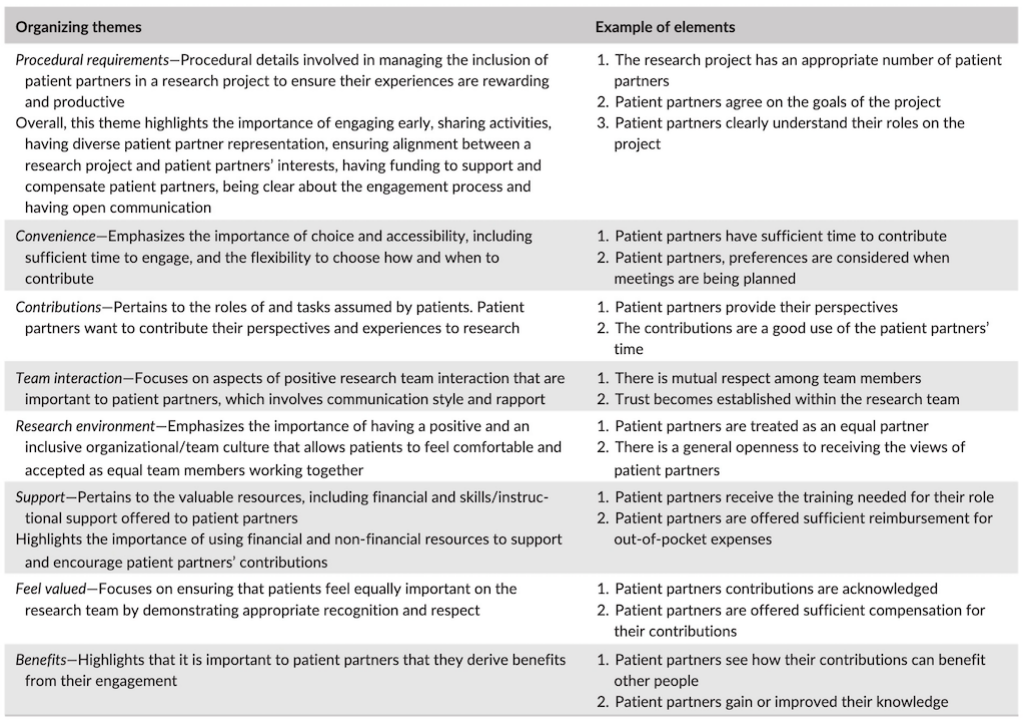
Organizing themes of the Patient Engagement in Research Framework with examples of corresponding elements (reprinted with permission from authors) [17].

There is a significant body of literature that helps conceptualize and operationalize the elements of patient engagement within this study [3,8,17-19]. Patient engagement is often considered a spectrum ranging from informing stakeholders to giving stakeholders complete decision-making autonomy. The intention for patient engagement in this project aligns with the term collaboration; wherein a partnership is formed, decision-making is a shared responsibility between the researchers and the patient group, and is inclusive of their knowledge, experience, and preferences. The process goal for our patient engagement approach is based upon identified metacriterion [8] of respect, trust, legitimacy, fairness, competence, and accountability in the development of knowledge. To operationalize this intent, we will use the Patient Engagement in Research (PEIR) framework [17] (Figure 1) to guide the actions and strategies of our patient engagement approach. Whereas the metacriteria help guide the goals of patient engagement, the PEIR framework highlights key themes that can be used as scaffolding for *how* to conduct meaningful patient engagement in research. Therefore, explicit planning and reporting of the patient engagement approach and activities within the project will be an important foundation of this study.

The purpose of this study is to (1) use patient engagement methods to establish a research collaboration with parents to cocreate a digital knowledge translation tool for parents caring for a child with functional constipation and (2) formally evaluate the patient engagement processes within this project to build the science of patient engagement in research.

## Methods

### Patient Engagement

This study forms part of a multistage research project to improve care and resources for families living with pediatric functional constipation (see diagnostic criteria in Multimedia Appendix 1) [20,21]. The preceding stage of qualitative, Interpretive Descriptive [22] research frames this proposed patient engagement phase and will be the primary recruitment source of our collaborators. The purpose of the qualitative research stage was to develop an in-depth understanding of parents’ experiences and information needs when caring for a child with functional constipation. Recruitment was through community and social media information posts shared in the summer and fall of 2019. Interested parents contacted the research team for further details. We recognize that parents who volunteer for such research are unlikely to reflect the general population, and we will explicitly cite this limitation in our findings. After sharing the information letter and discussing any questions, 18 parents consented and participated in semistructured interviews. After the interview, parents were asked if they would like to allow the research team to keep their contact information and be notified about the subsequent stage; patient-engagement to cocreate a digital knowledge translation tool.

The operationalization of patient engagement in this project is through the creation of a parent collaborator group and is detailed as follows. A parent collaborator group will be formed by inviting all participants from the qualitative portion of the research to move forward in a new role as a member of the parent collaborator group. Through collaboration, we will work together to establish priorities and cocreate a digital knowledge translation tool for parents caring for a child with functional constipation. This stage of the research fits within the *tailoring knowledge* portion of the Knowledge-to-Action (KTA) framework [23]. The patient engagement process and activities described in this stage are meant to provide a framework rather than a rigid protocol because the parent collaborator group has not been formed, and their contributions to shaping the research process are critical to upholding the legitimacy of parents’ collaborative role in this stage.

We did not locate any evidence to support best practice about the optimal group size for patient engagement in research. Instead, we will build the parent collaborator group based on practical considerations and recommendations of coauthors with extensive experience working with parent groups. Specifically, the size of the group should foster meaningful engagement. That is, we strive to develop a group that is large enough to be able to have a discussion, and everyone has the opportunity to share ideas. Conversely, we do not want a group so large that it is unmanageable. Lastly, we remain cognizant that these are parents with children, and they may not be able to attend every session, so we aim to have enough flexibility in our meetings to accommodate for all of these factors (online synchronous and asynchronous access). We anticipate a reasonable target size of between four and twelve members will be sufficient to build meaningful engagement. Although the primary source of collaborators in this stage will be from the preceding qualitative stage of the project, additional parents who have experience with childhood functional constipation will be welcomed to join the parent collaborator group as they become known to other members of the group (friends or community members known to have a child with functional constipation).

At our first meeting, detailed verbal and written information about the commitment required by the study will be provided. Informed consent will be sought from interested participants. Members may revoke their consent to participate at any time. The first meeting will be facilitated by a registered professional (psychologist or social worker) with extensive group facilitation experience to establish group norms and support an effective group process. Subsequent meetings will be cofacilitated by the researcher and parents. The aim of the project will be discussed, including the following key points. First, parent participation is explicitly being sought to ensure this project will accurately address the challenges and improve the experiences of families living with pediatric functional constipation. Second, parents will be supported to develop new skills if desired, but their experiential knowledge already qualifies them as valuable partners in this project. Third, parents will share decision-making responsibility with the researchers for the content, form, and style of the knowledge translation tool. Decision-making processes within the group will be documented and determined by the group. For example, the group may choose to use a modified Delphi technique [24] or focus on a robust discussion to generate consensus. Fourth, although individual input is desired, participation will also involve interacting with other parents affected by pediatric functional constipation. Fifth, differing perspectives amongst group members are expected and considered beneficial because the aim is to advocate for the needs of the larger parent community as a whole. That is, participants need not aim for unanimous agreement on topics of discussion. Finally, the concepts of respect, trust, legitimacy, fairness, competence, and accountability will be our guideposts for the work of the parent collaborator group.

Expectations for the activities and commitment of the parent collaborator group will also be discussed. The time commitment is based on previous experience of coauthors and is anticipated to be approximately one-hour meetings held every 3-4 weeks for 1-3 months. This timeline is flexible and will be adapted based on the progress and needs of the parent collaborator group. Meeting locations will be central to parents, accessible by public transportation, and include childcare and light refreshments. The content of the digital knowledge translation tool will stem from (1) best practice guidelines and clinical recommendations for the management of pediatric functional constipation, and (2) the themes and experiences generated from the qualitative inquiry of the preceding stage. The methods and process for developing the knowledge translation tool are based on existing literature [25-28] and previous experience with creating knowledge translation tools for parents. This research is situated within a larger program of research in a nationally funded knowledge mobilization network, Translating Emergency Knowledge for Kids (TREKK) [29], where a clinical team develops bottom line recommendations, developed by exploring practice guidelines and the best available synthesized research evidence. All bottom line recommendations are vetted through a large, clinical focused national committee. The format of the knowledge translation tool will be determined by the parent collaborator group while building on the strengths of a narrative-based medium. For example, previously successful knowledge translation tools have been whiteboard videos and digital storybooks. A graphic designer and creative writer will be available to support the development of a high-quality digital knowledge translation tool. The design team of the writer and graphic designer will be provided with a story outline that reflects the combined experiences and most salient themes from the qualitative inquiry. The parent collaborator group will work with the design team to revise and build the knowledge translation tool through iterations to address questions of clarity, potential bias or marginalizing factors, ease of use, relevance, and other factors as determined by the parent collaborator group. Upon completion of the knowledge translation tool, the final component of the project will be to evaluate the process of patient engagement in the project. Although not directly part of this stage of the research project, the knowledge translation tool (after completion) will be formally evaluated and tested for usability. The knowledge translation tool will also be made widely available on digital and social media platforms.

### Evaluation Design

The evaluation of the parent collaborator group will use a multimethod design with both quantitative and qualitative components. A multimethod design was chosen to answer two related but distinct research questions. First, the quantitative component will use the Public and Patient Engagement Evaluation Tool (PPEET) participant questionnaire [30,31], which includes survey questions with Likert response options to examine the question, “To what degree did the patient engagement processes of the research meet the intended meta-criterion of respect, trust, legitimacy, fairness, competence, and accountability [8]?” The qualitative component will use open-ended questions to explore in more detail, “Why or how did/didn’t the patient engagement processes of this research project meet the meta-criterion?” The rationale for using quantitative and qualitative methods in this stage of the research aligns with the purpose of *expansion* or *enhancement* by using an additional method to augment and further detail the findings [32,33]. Due to the focused nature of the evaluation and the small size of the parent collaborator group, both the quantitative and qualitative aspects of the study will be limited to descriptive methodologies.

### Sample

All caregivers who participate in the parent collaborator group will be invited to participate in the evaluation phase. Parents who did not continue for the full duration of the project will also be included in the sample if they are willing. Parents who were invited to participate in the group but declined will be asked if they are willing to share any feedback about what may have influenced their decision not to join the group.

### Data Collection

Data collection will occur after the completion of the knowledge translation tool development. The PPEET patient questionnaire [30,31] will be copied into a digital format by entering the questions and response fields into the secure surveying platform SimpleSurvey. Parents will receive digital access to the questionnaire, which can be completed anonymously. Demographic questions which are considered indirect identifiers will be optional data fields. The survey instructions will include an explanation that if the demographic questions are answered, the respondent’s data will remain confidential but may no longer be anonymous (to the researchers). The tool aims to generate data concerning the key features of the engagement approach and the participants’ perceptions of impact [31]. The PPEET includes 14 survey questions with five Likert-scale response options ranging from strongly agree to strongly disagree. The tool includes open-ended questions querying how the results may be used, the best aspect of the engagement, and areas for improvement. Qualitative analysis will be used to understand the open-ended question portion of the PPEET to generate more in-depth data. Documents from the parent collaborator group meetings such as agendas, minutes, and decision processes will be used as additional data sources to more fully answer the research questions.

### Analysis

The two types of data collected will be analyzed and reported separately. The findings from the quantitative and qualitative data will be integrated into the discussion if there are sufficient commonalities and inter-relationships.

Data from the Likert-scale questions will be entered into SPSS version 25. Descriptive statistics will be used to report group characteristics and question responses, including mean, median, and/or the mode (as appropriate), and range (or IQR, as appropriate). Frequency and percentages will be reported for categorical demographic information. No further analysis is planned because there is no comparative element of the design.

We will use directed content analysis [34] to explore participant responses relative to the themes of the PEIR Framework [17] using a combination of deductive and inductive analyses. Documents from parent collaborator group meetings (agendas, minutes, decision processes) will also be used as data sources for qualitative analysis. Data will be cleaned and transferred into NVIVO version 11 (QSR International). All responses will be categorized according to the PEIR framework codes: *procedural requirements*, *convenience*, *contributions*, *support*, *team interaction*, *research environment*, *feeling valued*, and *benefits* [17]. Text that cannot be coded into one of these categories will be coded with another label that captures the meaning of the response. Finally, we will compare the extent to which the data fit within the PEIR framework versus other themes. Interested members of the parent collaborator group will also be invited to contribute to the analysis and dissemination of the evaluation findings in order to maintain engagement in the collaborative relationship. The manuscript produced from this stage of the research will include reporting each element described by the Good Reporting of A Mixed Methods Study criteria [35].

### Ethics

Approval from the appropriate University Health Research Ethics Board is complete for this project (#Pro00087548). Each participant will receive an information sheet that will provide details on the purpose of the study, identify the potential risks/benefits, and explain the voluntary nature of their participation. Participants may choose not to answer particular questions and can revoke consent from participating in the parent collaborator group at any time. Evaluation data will be collected anonymously; therefore, individual participant data cannot be removed after it is collected. Data will be kept confidential, except for the duty to report any information relating to child welfare. Any information disclosed that falls under mandatory reporting laws (eg, safety and well-being of a child) would be shared first with the disclosing participant. Eligible participants will receive a written consent form to be read and signed before enrolling in the study. All data will be stored using secured software on a password-protected server.

### Data Management

Survey data will be collected on participants’ computer or tablet devices through the SimpleSurvey platform. SimpleSurvey is a secure online platform with secure servers in Canada, protected by several firewalls and three physical layers of security. Data collected through the online platform is completely anonymous and cannot be traced back to any one individual. The data is stored on SimpleSurvey servers until data collection for the specific survey/project is complete. Once data is downloaded onto the University of Alberta servers, it will be deleted from SimpleSurvey storage. Data will be stored on a secure drive, which is hosted by the University of Alberta, Faculty of Nursing, secure server system. The server is backed up twice a day. Files can be recovered if accidentally deleted/lost/corrupt. In the event of system-wide corruption, an external hard drive is used to back up the data once a month. This hard drive is kept in a locked area within a locked office.

## Results

Recruitment for the parent collaborator group is planned for June 2020. Once the group is formed, the development of the digital knowledge translation tool for parents caring for a child with functional constipation is expected to take 3-4 months. Data collection for the evaluation of patient engagement processes will occur when the digital knowledge translation tool has been built and is expected to take 2-4 weeks to optimize the number of responses. The results of this study are expected to be published by the end of 2020.

## Discussion

This study will include the development of a relevant and accessible digital knowledge translation tool created *with* and *for* parents caring for a child with functional constipation. The findings will also fill gaps in the evidence supporting the processes of patient engagement in research. Our reported patient engagement processes are widely applicable, meaning others can use this protocol to guide patient engagement and evaluation in a variety of contexts. Specifically, the results can inform future research collaborations to ensure that contributions by patient stakeholders are optimized, and challenges recognized and planned for accordingly. For example, avoiding tokenism, fostering inclusivity, and building capacity are knowledge gaps within patient engagement methods in research that may be better understood through widespread evaluations and dissemination. The results of this study can help build the science of patient engagement in research. Limitations of the study and findings will be discussed. Despite our planning and intentions, this study may face challenges such as small sample size or significant attrition. We commit to full disclosure of the barriers encountered and the potential implications for the results. Given the emergent nature of PE evaluation, we suggest that studies with negative or limited findings are equally important to understand the barriers to further development of this field.

This study fits within the KTA framework [23] as a component of *tailoring knowledge* by creating a knowledge translation tool. Future projects related to this research will plan and examine the integration of the knowledge translation tool into the *action cycle* of the KTA framework [23]. For example, assessing usability by a broader audience contributes to adapting the knowledge to the local context and can also help identify potential barriers to use. In addition to the creation of a digital, patient-direct knowledge translation tool, knowledge translation activities will be woven throughout this research. Specifically, the topic of functional constipation aligns with priority areas of research identified by a national needs assessment of care providers; therefore, the foundation for this research stems from an existing relationship with clinical knowledge stakeholders. The use of a patient engagement approach in this research allows for explicit and ongoing inclusion of stakeholders; thus, integrating end-users of the knowledge into the development processes. Lastly, the dissemination of the findings from this study will include tailored presentations to stakeholder groups and manuscript publication to target healthcare researchers.

## Appendix

Multimedia Appendix 1 Diagnostic criteria for functional constipation.

## Abbreviations

KTA: Knowledge-to-Action
PEIR: Patient Engagement in Research
PPEET: Public and Patient Engagement Evaluation Tool
